# Data obtained with a novel approach to estimate installment loan acquisition costs

**DOI:** 10.1016/j.dib.2018.04.027

**Published:** 2018-04-12

**Authors:** Onyumbe Enumbe B. Lukongo, Thomas W. Miller

**Affiliations:** a Southern University and A & M College, Baton Rouge, LA, USA; b Mississippi State University, Mississippi State, MS, USA

**Keywords:** Data, Loan acquisition costs, Loan desert, Interest rate cap, Arkansas

## Abstract

The data presented in this study were obtained from a novel approach to estimate a comprehensive loan acquisition cost. The latter includes commute costs and wage losses in addition to the monthly installment payments. These cost estimates represent the monetary value (in U.S. dollars) of the costs of driving to and from the installment lender storefront and that of the potential hourly wage losses, that is, wage loss from the driving time and the time spent at the loan office filling out the required paperwork to obtain the loan. Borrowers only get the net loan proceeds, that is, the original loan amount minus the comprehensive loan acquisition costs. The study area has 160 counties. It was created from the ESRI ArcGIS Map (a mapping software) using the spatial data from the U.S. Census, Topologically Integrated Geographic Encoding and Referencing (TIGER) Cartographic boundary files representing the geographies of states and counties. Using the U.S. road networks, the origin of the trip is a county seat in Arkansas and the destination of the trip is a county seat in a surrounding state of Tennessee, Mississippi, Louisiana, Texas, Oklahoma, and Missouri. The transportation networks were established using Google Earth/Directions to efficiently measure the travel time (distance). The average cost of a trip of 17 cents (U.S. dollar) was calculated based on the U.S. Department of Transportation Survey data, which identify important attributes of a typical vehicle used in a county such as model make, age of the vehicle, fuel consumption, etc. There are 10 occupational industry sectors where a typical borrower has a job. To estimate wage loss, the data were gathered from the U.S. Department of Labor, Bureau of Labor Statistics, namely, the Occupational Employment Statistics. Putting the missing pieces together, the data contain in this study improve our understanding of extra costs borne by borrowers located in the “loan desert” area. As expected, interior counties post high loan acquisition costs compared with border counties. The data from this study are useful to the public, businesses, policymakers, and researchers working on consumer finance.

**Specifications Table**

**Table:** 

Subject area	Economics, Financial markets
More specific subject area	Economics of consumer finance
Type of data	Table, figure, Excel file, text
How data was acquired	Data were obtained from the U.S. Bureau of Labor Statistics, Google Earth, U.S. Department of Transportation
Data format	Raw, estimated
Experimental factors	
Experimental features	Secondary data on wages by occupational industry, commute costs
Data source location	
Data accessibility	The data are provided in this article; http://www.bls.gov/oes/current/oes_26300.htm
Related research	

**Value of the data**

● It is the first attempt in the literature to estimate a comprehensive loan acquisition cost, which includes commute costs and wage losses in addition to the monthly installment payments.
● Cost estimates are presented based on the occupational industry sector where a typical borrower has a job.
● It emphasizes the notion that the interest rate cap is unprofitable for businesses and imposes extra costs to borrowers as evidenced by “loan desert” in the interior of Arkansas.
● It is useful to the general public, businesses, policymakers, and researchers working on consumer finance.

## 1 Data

The detailed dataset is reported in the “Loan Acquisition Costs” MS Excel file, which summarizes the average loan acquisition costs for 10 occupational industry sectors (Natural resources and mining; Construction; Manufacturing; Trade, transportation, and utilities; Information; Financial services; Professional and business services; Educational and health services; Leisure and hospitality; and Other services) where a typical borrower has a job. Wages associated with each occupational industry sector are presented in the “Wages” MS Excel file. One of the requirements for loan application packet is that a representative borrower shall present a recent pay stub.

**Fig. 1:**
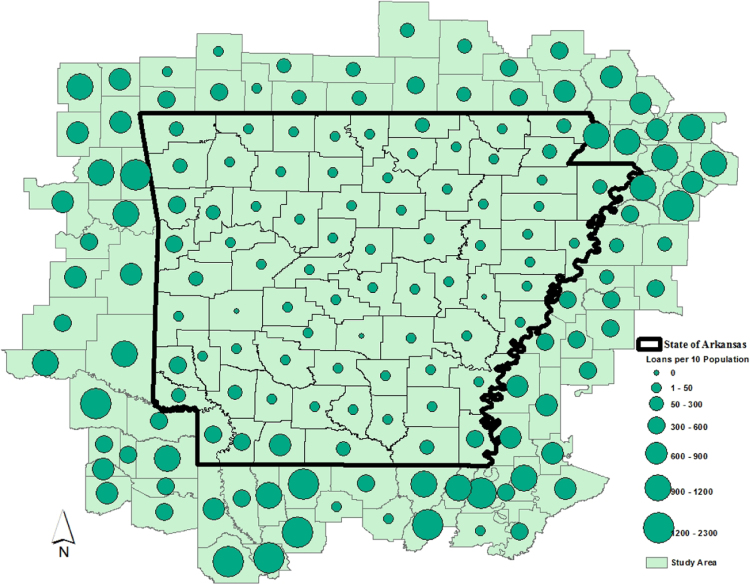
Loans per 10,000 population in Arkansas and Two-Nearest Counties from its Surrounding States.

**Fig. 2:**
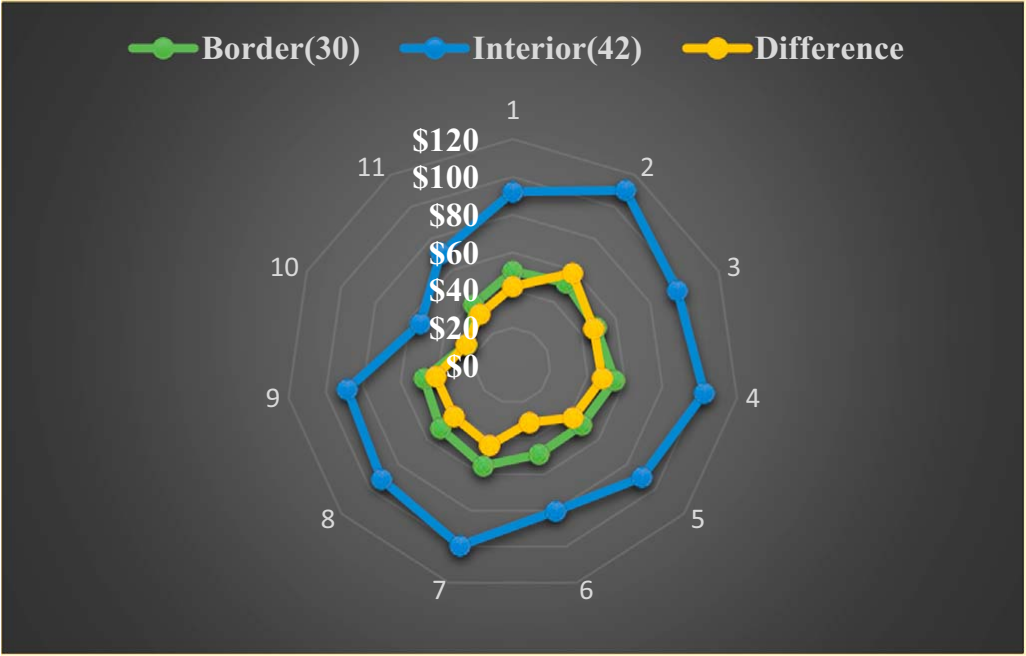
Average Loan Acquisition Costs ($) in Border and Interior Counties, Arkansas by Occupational Industry Sector Note: the following figures represent different occupational industry in relation to average loan acquisition costs ($), 1 = Total, all industries; 2 = Natural resources and mining; 3 = Construction; 4 = Manufacturing; 5 = Trade, transportation, and utilities; 6 = Information; 7 = Financial activities; 8 = Professional and business services; 9 = Education and health services; 10 = Leisure and hospitality; 11 = other services.

**Fig. 3:**
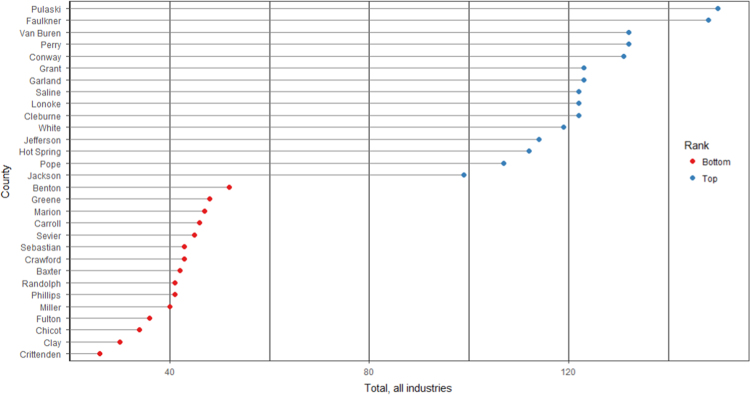
Loan acquisition costs for top and bottom fifteen counties, all industries.

**Fig. 4:**
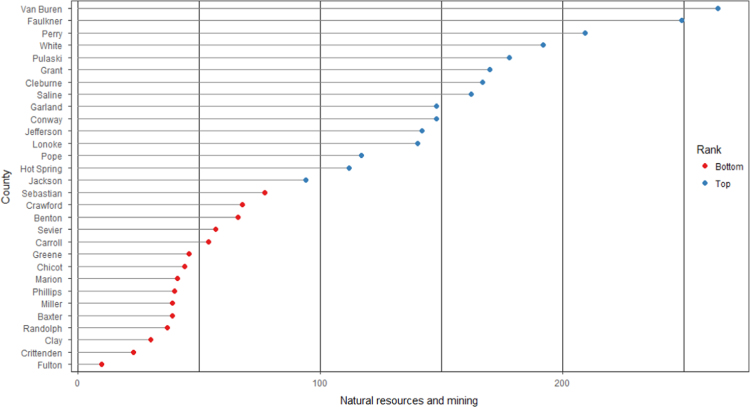
Loan acquisition costs for top and bottom fifteen counties, natural resources and mining industry.

**Fig. 5:**
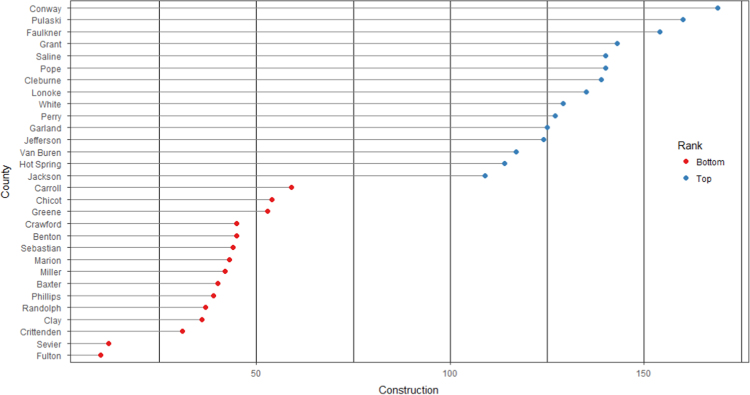
Loan acquisition costs for top and bottom fifteen counties, construction industry.

**Fig. 6:**
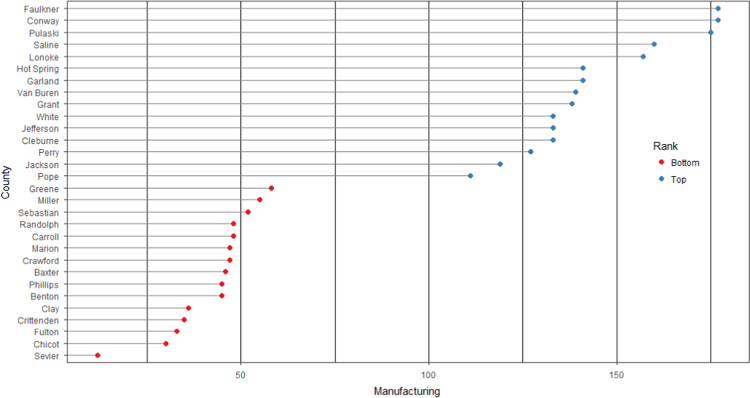
Loan acquisition costs for top and bottom fifteen counties, construction industry.

**Fig. 7:**
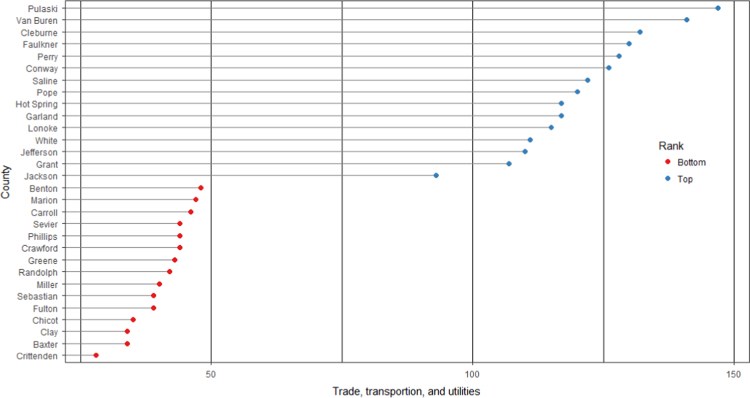
Loan acquisition costs for top and bottom fifteen counties, trade, transportation, and utilities industry.

**Fig. 8:**
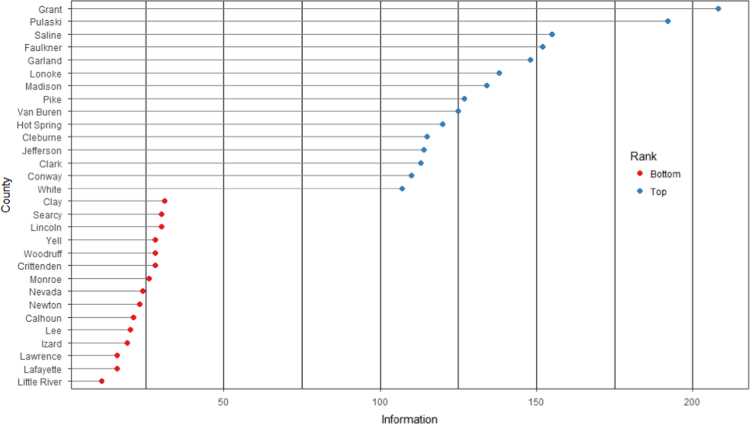
Loan acquisition costs for top and bottom fifteen counties, information.

**Fig. 9:**
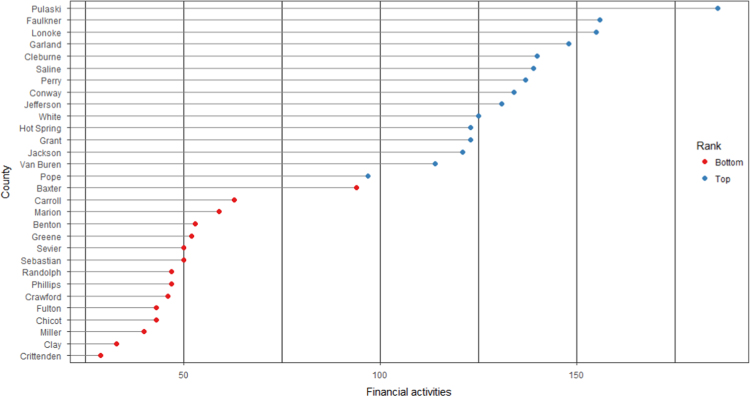
Loan acquisition costs for top and bottom fifteen counties, financial activities industry.

**Fig. 10:**
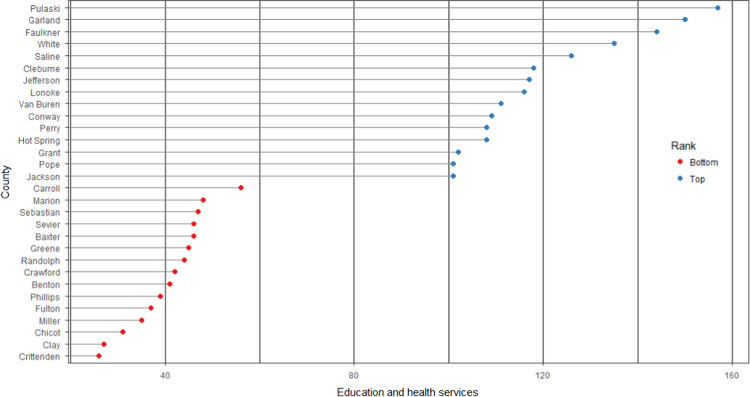
Loan acquisition costs for top and bottom fifteen counties, education and health services industry.

**Fig. 11:**
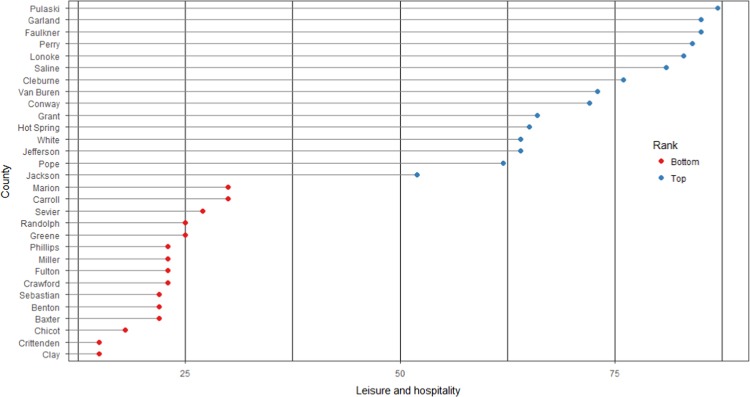
Loan acquisition costs for top and bottom fifteen counties, leisure and hospitality industry.

**Fig. 12:**
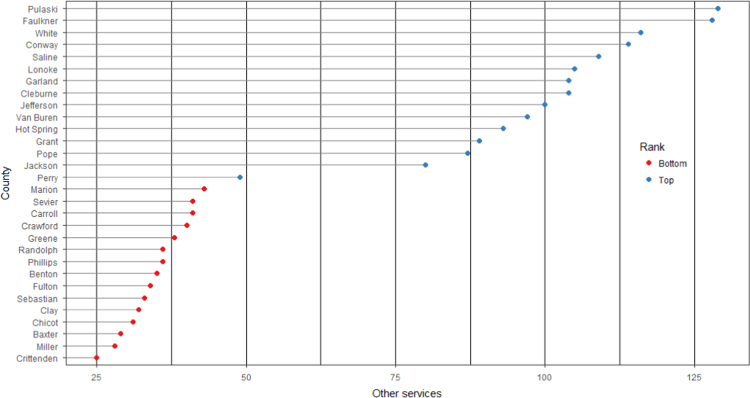
Loan acquisition costs for top and bottom fifteen counties, other services industry.

The average loan acquisition costs are presented based on the relative location of counties with respect to Arkansas borders, that is, border and interior counties. Fig. 1 displays loan usage per 10,000 population organized in seven classes, 0; 1–50; 50–300; 300–600; 600–900; 900–1200; 1200–2300. It should be noted that the size of the dot on the map is comparative to the loan usage. Fig. 2 shows the average loan acquisition costs ($) in border and interior Counties, Arkansas by occupational industry sector. A detailed account is provided in the “Loan Acquisition Costs” MS Excel file along with the results from the means comparison test using the *t*-statistic evaluated at the conventional levels of significance of 1%, 5%, and 10%. Results suggest very strong evidence of the difference of loan acquisition costs between border and interior counties. In addition, Fig. 3, Fig. 4, Fig. 5, Fig. 6, Fig. 7, Fig. 8, Fig. 9, Fig. 10, Fig. 11, Fig. 12 stack the top fifteen counties or the highest loan acquisition costs all located in the interior Arkansas and the bottom fifteen counties or the lowest loan acquisition costs all located in the borders of Arkansas.

## 2 Experimental design, materials and methods

### 2.1 Importance of data

In the early 1900s, a new loan source emerged through the collaboration of lenders and consumer advocates. Their recommendations were captured in the Uniform Small Loan Law (USLL) of 1916. By 1940, all but nine states had adopted some version of this model legislation regulating cash installment loans.

The Arkansas legislature chose to keep the constitutionally imposed rate cap of ten percent. In November 2010, however, amendment 89 to the Arkansas constitution passed with about 64% of the votes cast supporting it. This amendment raised the maximum interest rate for all loans, including cash installment loans, from 10% to 17%.

An Annual Percentage Rate (APR, thereafter) of 17% has not induced small dollar cash installment lenders to operate in Arkansas. Revenues on loans with this cap do not cover loan production and servicing costs. Each of the six states (Missouri, Tennessee, Mississippi, Louisiana, Texas, and Oklahoma) that border Arkansas have no rate cap, or a rate cap much higher than Arkansas. As a result, cash installment loans are offered by finance companies in each of these six states.

A cash installment loan 1) has equal payments that fully amortize the debt after the borrower makes the last payment, and 2) has payments consisting of interest and an amount that reduces the principal owed. In these two ways, cash installment loans are like the familiar installment loans made to finance the purchase of appliances, furniture, or vehicles. Unlike these sales finance products, borrowers can use the proceeds from a cash installment loan in any manner they wish.

Previous spatial analysis studies on small dollar loans focus on payday loans, which are short-term, lump-sum loans. Studies examine the locations picked by payday lenders [1], [2]; examine the effects of payday loans on the indebtedness of veterans, military personnel, financially distressed families, and poor workers [3], [4]; document how payday lenders provide a way for consumers to weather a cash shortfall [5], or examine how consumers substitute among small dollar loan products [6].

By contrast, we examine small dollar cash installment loans. The specific contribution of this study is to provide a novel way to estimate the total acquisition costs of a cash installment loan by including (i) the commuting costs of driving to and from the installment lender; and (ii) the potential hourly wage losses for the driving time and the time spent at the loan office, filling out the required paperwork to obtain the loan. Borrowers only get the net loan proceeds, that is, the original loan amount minus the loan acquisition costs.

### 2.2 Methodology

For convenience, we assume that the origin of each round trip is an Arkansas county seat and the destination is a county seat in another state. The standard practice in the spatial analysis is to use a built-in tool in the ArcGIS Map software, the Euclidean distance, to measure the distance between the origin and the destination. This standard practice fails to account for different landscapes, hills, valleys, and other natural barriers such as bodies of water. To address these limitations, we use the travel time in minutes and distance in miles from the Google Earth/Get Directions application.

Then, we build a table of origin (county seats in Arkansas) and destination (county seats in two nearest counties of the bordering states). We employ a least cost approach using the minimum travel time between each origin/destination pair. We obtain a vehicle operating cost of $0.17, as detailed in [7]. We obtain the average hourly wage in industrial sectors from the U.S. Bureau of Labor Statistics’ Quarterly Census of Employment and Wages as the 3rd quarter of 2013 [8]. The industrial sectors consist of firms organized in goods-producing industrial sectors (natural resources and mining; construction; manufacturing) and service-providing sectors (trade, transportation, and utilities; information; financial activities; professional and business services; education and health services; leisure and hospitality; and other services).

The proposed installment loan acquisition costs (*LAC*_*ij*_) from county-seat *i* in Arkansas to the closest county-seat *j* in any of the six surrounding states measures the overall costs of a loan is the sum of the commute cost and the foregone wages for the loan borrower, by industry classification. That is:

(1) $$LAC_{ij}=TCC_{ij}+VT_{i}\cdot t_{ij}+VPWT_{i}$$

where *TCC*_*ij*_ represents the total commute costs expressed in dollars for the minimum travel time; *VT*_*i*_ is the value of time for a consumer of county-seat *i* captured by the average hourly wage in industry sector, and *t*_*ij*_ is the minimum travel time between two county seats located in Arkansas and in any county seats of surrounding states; and, *VPWT*_*i*_ equals one hour of foregone wages from completing the paperwork at the installment loan storefront.

Related to the spatial analysis, a thematic map (Fig. 1) was designed and produced following the standard practice in geographic information systems and cartography. Once the end product has been defined, that is, the map, the following task is conducted in four steps. The first step consists of acquiring the geographic referenced file, which is also known as a shapefile from the U.S. Census TIGER files. The second step is to define the appropriate projection of the study area. The third step deals with the delimitation of the study area using a spatial query select by location. The fourth step merges the installment loans and the georeferenced data using the function join-and-relate in the ESRI ArcGIS Map package. Given that the data arrangement has been completed, the next task is to produce the final map in three steps. The application of cartography goodies, which is the selection of seven classes to summarize the results, and that of the symbology shown in the legend, and the insert of the orientation arrow pointing to the north.

Finally, we test the hypothesis of no-difference between the average loan acquisition costs ($) in border and perimeter counties using a formal mean-comparison *t*-test and its estimated value (see MS Excel file) is evaluated at 1%, 5%, and 10% significance levels, respectively.

### 2.3 Geospatial and statistical analyses of loan acquisition costs

A thematic ArcGIS map (see Fig. 1) portrays the distribution of cash installment loans across the study area. It clearly shows the installment loan desert in the interior counties of Arkansas. A solid black line shown in Fig. 1 indicates the boundaries of the state of Arkansas relative to two neighbor counties in the states of Missouri, Tennessee, Mississippi, Louisiana, Texas, and Oklahoma when reading the figure clockwise. A detailed account of the data for the top and bottom fifteen countries is provided in Fig. 3, Fig. 4, Fig. 5, Fig. 6, Fig. 7, Fig. 8, Fig. 9, Fig. 10, Fig. 11, Fig. 12. *R* package was employed to create Fig. 3, Fig. 4, Fig. 5, Fig. 6, Fig. 7, Fig. 8, Fig. 9, Fig. 10, Fig. 11, Fig. 12.

Fig. 2 presents the average loan acquisition costs for both interior and perimeter counties and the results of the mean-comparison tests (interior vs. border counties) and their related mean-comparison *t*-statistics evaluated at 1% significance level. Data suggest high loan acquisition costs in interior counties (in blue) compared to border counties (in green) with low loan acquisition costs.

Fig. 3, Fig. 4, Fig. 5, Fig. 6, Fig. 7, Fig. 8, Fig. 9, Fig. 10, Fig. 11, Fig. 12 present the loan acquisition costs for the top fifteen (all interior counties) and bottom fifteen (all border counties) by occupational industry sector. The *y*-axis shows the top and bottom fifteen counties based on the loan acquisition costs and the *x*-axis shows both the occupational industry sector and the loan acquisition costs expressed in U.S. dollars.

### 2.4 Summary of the data

Data are consistent across occupational industry sectors. The average amount borrowed by Arkansas residents is about $1051 [7]. Consequently, loan acquisition costs are significant.

We propose and implement a novel way to estimate the total acquisition cost for Arkansas borrowers traveling out of state to obtain a cash installment loan. Overall, data show that it is expensive for Arkansans who reside in the interior counties to acquire small dollar installment loans. We study this expense across different occupational industry sectors. The data lend support to the notion that a small dollar cash installment loan desert exists in the interior counties of Arkansas. The complete table of loan acquisition costs data is provided in the MS Excel “Loan Acquisition Costs.xls” file along with wages data and the loan acquisition cost means comparison results.

## Funding

The authors received no financial support for the research, authorship, and/or publication of this article.
