# Supplementary material for: Data obtained with a novel approach to estimate installment loan acquisition costs
Source: Data Brief. 2018 Apr 12;18:1257–66. doi: 10.1016/j.dib.2018.04.027 (PMC5997097; doi:10.1016/j.dib.2018.04.027)
Supplement: Supplementary file 1 — Transparency document [file mmc1.docx]

Baton Rouge, LA, USA 4/03/2018

To: The Managing Editor

Data in Brief

Elsevier

RE: **Declaration of Conflicting Interests and Funding**

The authors declared no potential conflicts of interest with respect to the research, authorship, and/or publication of this article.

The authors received no financial support for the research, authorship, and/or publication of this article.

Corresponding Author

Dr. Lukongo
